# CCL4 participates in the reprogramming of glucose metabolism induced by ALV-J infection in chicken macrophages

**DOI:** 10.3389/fmicb.2023.1205143

**Published:** 2023-06-02

**Authors:** Huixian Wu, Gul Zaib, Huan Luo, Wang Guo, Ting Wu, Shutong Zhu, Chenjun Wang, Wenxian Chai, Qi Xu, Hengmi Cui, Xuming Hu

**Affiliations:** ^1^Institute of Epigenetics and Epigenomics, College of Animal Science and Technology, Yangzhou University, Yangzhou, Jiangsu, China; ^2^Jiangsu Key Laboratory for Animal Genetic, Breeding and Molecular Design, College of Animal Science and Technology, Yangzhou University, Yangzhou, Jiangsu, China; ^3^College of Veterinary Medicine, Yangzhou University, Yangzhou, Jiangsu, China; ^4^Joint International Research Laboratory of Agricultural and Agri-Product Safety, Ministry of Education of China, Yangzhou University, Yangzhou, Jiangsu, China; ^5^Changzhou Animal Disease Prevention and Control Center, Changzhou, Jiangsu, China

**Keywords:** avian leukosis virus, CCl4, macrophages, glucose metabolism, immunity

## Abstract

Interferon and chemokine-mediated immune responses are two general antiviral programs of the innate immune system in response to viral infections and have recently emerged as important players in systemic metabolism. This study found that the chemokine CCL4 is negatively regulated by glucose metabolism and avian leukosis virus subgroup J (ALV-J) infection in chicken macrophages. Low expression levels of CCL4 define this immune response to high glucose treatment or ALV-J infection. Moreover, the ALV-J envelope protein is responsible for CCL4 inhibition. We confirmed that CCL4 could inhibit glucose metabolism and ALV-J replication in chicken macrophages. The present study provides novel insights into the antiviral defense mechanism and metabolic regulation of the chemokine CCL4 in chicken macrophages.

## Introduction

The innate immune system acts as a first line of defense against invading microbial pathogens and launches two general antiviral programs in response to viral infections. One antiviral response is mediated by the induction of type I and III interferons and the subsequent upregulation of IFN-stimulated genes (ISGs), which play a pivotal role in delaying the replication of the virus in the initial stage of virus infection (Lazear et al., 2019; Blanco-Melo et al., 2020). The other antiviral response regulates and determines specific subsets of leukocytes, such as inflammatory monocytes, by chemokine secretion to help eliminate/eradicate the virus. These broad antiviral responses have accelerated virus evolution to counter interferon and chemokine-mediated antiviral responses. Viruses have evolved multiple strategies of interferon evasion to establish successful replication cycles in their hosts (Ten, 2017). In addition to antagonizing the interferon system, many oncogenic viruses also hijack chemokine systems to manipulate the replication of infected cells (Damania, 2004).

Presently, the innate immune system has emerged as a prominent player and paves our understanding of systemic metabolism (Cortese et al., 2014; Li et al., 2018; Jung et al., 2019; Shen et al., 2021; Wang et al., 2022). In addition to evading the innate immune system, viruses usually interfere with host metabolism. Viruses rely entirely on the host metabolic machinery to complete their life cycle and have evolved to rewire host cell glucose and glutamine metabolism (Thai et al., 2015). Several studies have demonstrated that the innate immune system regulates glucose metabolism. For example, IFN-γ negatively affects glucose metabolism (Greer et al., 2016). IFN-γ can restrain host cell glycolysis (Shima et al., 2018), and this metabolic reprogramming is a key component of classical inflammatory macrophage activation (Su et al., 2015). Type I interferons have also been shown to reprogram metabolism (Lercher et al., 2019, 2020). IFN-β prevents the shift to aerobic glycolysis in inflammatory macrophages and restrains macrophage metabolism (Olson et al., 2021). Previous research has also highlighted that chemokines regulate glucose metabolism, including C-C motif chemokine ligand 4 (CCL4) and CCL5 (Chou et al., 2016; Gao et al., 2016; Ajoy et al., 2021; Chang and Chen, 2021). The co-receptor of CCL4 and CCL5, C-C chemokine receptor 5 (CCR5), has been demonstrated to restrain aerobic glycolysis (Blanco et al., 2021) and to contribute to systemic insulin sensitivity and glucose metabolism (Chou et al., 2016). Therefore, viruses may ultimately have a significant impact on host metabolism by modulating the innate immune system.

However, few studies have demonstrated the important communication between the immune system, virus infection, and metabolism. CCL4, previously known as macrophage inflammatory protein (MIP)-1β, belongs to the CC chemokine subfamily and has important chemokinetic and inflammatory functions through interaction with the chemokine receptor CCR5 (Mukaida et al., 2020). Recently, CCL4 has been interrelated and linked with genetic resistance and the chicken’s response to infection by avian viruses. High expression of the CCL4 gene has been shown to negatively affect susceptibility to NDV infection. Previous studies have signified and deciphered the role of CCL4 in chickens. For example, AIV-induced CCL4 in chicken lung tissues (Ranaware et al., 2016) was higher in AIV-resistant chickens than in AIV-susceptible chickens (Vu et al., 2021). CCL4 was also upregulated in the IBDV-infected chicken lines P and N, with a greater increase seen in the most susceptible line, line P (Mohd Isa et al., 2020); this upregulation of CCL4 indicates a pro-inflammatory response, which is correlated with the bursal immunopathology of IBDV.

The current study addressed whether the established dialog between the chemokine CCL4 and glucose metabolism is involved in virus infection. We selected an important viral model of avian leukosis virus subgroup J (ALV-J) in chicken. ALV-J is an alpha-retrovirus and causes tumors and immunosuppression in chickens, and there is no effective vaccine against ALV-J (Venugopal, 1999; Payne and Nair, 2012). We found that the chemokine CCL4 is negatively regulated by glucose metabolism and ALV-J infection in chicken macrophages. We confirmed that CCL4 could inhibit glucose metabolism and ALV-J replication in chicken macrophages. The present study indicated that the chemokine CCL4 might play a key role in antiviral defense and the reprogramming of glucose metabolism after ALV-J infection in chicken macrophages.

## Materials and methods

### Cell culture

The chicken macrophage-like line HD11 was obtained from the Laboratory of Avian Preventive Medicine, Yangzhou University, China. HD11 cells were derived from chicken bone marrow and transformed with the avian myelocytomatosis virus MC29 (Beug et al., 1979). The chicken macrophage cell line HD11 was maintained in DMEM-high glucose or DMEM-low glucose (Gibco, USA) with 10% FBS at 41°C, 5% CO_2,_ and 95% humidity.

### Viral infection

The virus strain JS09GY3 (GenBank accession number GU982308) was isolated from field-infected commercial layer chickens with haemangioma, myeloid leukosis, and insertion in the E element (Wu et al., 2010). This virus strain induced robust immune responses in the chicken bursa of Fabricius (Hang et al., 2014) and was selected to study the antiviral function of CCL4. HD11 cells were seeded into six-well plates and infected with the JS09GY3 strain of ALV-J at a multiplicity of infection (MOI) of 1 and 5. At 6, 12, 24, and 36 h post-infection (hpi), cells were collected for ALV-J replication and host gene expression analysis.

### Drug treatment

We used two drugs, 2-deoxy-D-glucose (2-DG) and sodium oxamate, to investigate the influence of glucose metabolism inhibitors on the expression of the chemokine CCL4 in HD11 cells. 2-DG is a glucose analog that acts as a competitive inhibitor of glucose metabolism, inhibiting glycolysis *via* its actions on hexokinase (Vander Heiden, 2011; Galluzzi et al., 2013; Zhang et al., 2014). Sodium oxamate, a specific lactate dehydrogenase A (LDHA) inhibitor, is a derivative of pyruvate that inhibits the conversion of pyruvate to lactate *via* lactate dehydrogenase, thus disrupting glycolysis (Zhang et al., 2019). HD11 cells were seeded into six-well plates, treated with the glycolysis inhibitor 2-DG or a specific LDHA inhibitor sodium oxamate for 36 h, and then collected for host gene expression analysis.

### MTT and CCK-8 assays

MTT and CCK-8 assays were used to evaluate the cytotoxicity on HD11 cells of high or low-glucose treatment, the glycolytic inhibitor 2-DG treatment, and the LDHA inhibitor sodium oxamate. MTT and CCK-8 assays were performed using MTT reagents and Cell Counting Kit-8 (Beyotime, China) following the ‘manufacturer’s instructions. All MTT and CCK-8 assays were performed in sextuplets and repeated in three independent experiments.

### Plasmid construction

The full-length CDS sequence of chicken CCL4 was obtained from the total RNA isolated from HD11 cells by RT-PCR using specially designed primers. The Hind III and Bam HI restriction sites were incorporated into the forward and reverse primers as follows:

ch-CCL4 forward primer 5′-CCC **AAGCTT** ATGAAGGTCTCTGTGGCTG-3′, and ch-CCL4 reverse primer 5′- CGC **GGATCC** TCAGTTCAGTTCCATCTTG-3′. The chicken CCL4 gene was then inserted into the eukaryotic expression vector pcDNA3.1 by double digested with Hind III and Bam HI restriction enzymes and confirmed by DNA sequencing. The pcDNA3.1 (+) vector was purchased from Invitrogen and stored in our laboratory.

### Transfection

HD11 cells were transfected with control or CCL4 using HighGene Transfection Reagent (ABclonal, United States) for 48 h, and total RNA and protein were collected for gene expression analysis. For viral infection experiments, HD11 cells were first infected with the ALV-J virus at a multiplicity of infection (MOI) of 5 for 12 h. Cells were then transfected with CCL4 or control using HighGene Transfection Reagent (ABclonal, United States) for another 36 h and then collected for ALV-J replication analysis.

### RNA interference assays

For the knockdown of the chicken CCL4 gene, HD11 cells were transfected with CCL4 or control siRNA using HighGene Transfection Reagent (ABclonal, United States) for 48 h. Then total RNA and protein were collected for gene expression analysis. HD11 cells were first infected with the ALV-J virus for viral infection experiments at an MOI of 5 for 12 h. Cells were then transfected with control or chicken CCL4 siRNA using HighGene Transfection Reagent (ABclonal, United States) for another 36 h and then collected for ALV-J proliferation analysis.

### Reverse transcription-quantitative PCR

RT**–**qPCR assays were performed according to previous studies (Chen et al., 2019). Briefly, total RNA was extracted from chicken cells or tissues using RNA-easy™ Isolation Reagent (Vazyme, Nanjing, China) according to the ‘manufacturer’s recommendations. The gDNA Eraser-treated RNA samples were reverse-transcribed with RT primers at 37°C for 15 min with HiScript III RT SuperMix for qPCR (+gDNA wiper) (Vazyme, Nanjing, China). Quantitative PCR was then performed with gene-specific primers and SYBR qPCR Master Mix (Vazyme, Nanjing, China) on a CFX Connect™ Real-Time PCR Detection System (Bio-Rad, California, United States). The GAPDH RNA levels were used as internal controls to normalize gene expression. The gene-specific primers sequences for ALV-J and GAPDH were referenced in previously published manuscripts (Chen et al., 2019). The other primers are designed by Primer Premier 6.0 software and described in Table 1.

**Table 1:** Primers used in this study.

Primer name	Nucleotide sequence 5′-3’	Accession number
CCL4 fwd	GTCCTCCTCATTGCCATC	NM_204720.3
CCL4 rev	TCAGTTCAGTTCCATCTTGT	
IFN-β fwd	GCCCACACACTCCAAAACACTG	NM_001024836.2
IFN-β rev	TTGATGCTGAGGTGAGCGTTG	
GLUT1 fwd	AGGAGATGAAGGAGGAGAG	NM_205209.2
GLUT1 rev	GACGATTGCGATGAGGAT	
GLUT3 fwd	GGCATAGTTGTAGGCATCC	NM_205511
GLUT3 rev	TTCTTCCTCCATCTTGTTGA	
HK1 fwd	CCGTGCCGACAATCTAAG	NM_204101.2
HK1 rev	AGGTCATCATAGTGCCAAC	
HK2 fwd	ATGGAGGAGATGAGGCAC	NM_204212.2
HK2 rev	GGTCCTGATGTCGTTGAG	
PFKL fwd	CGATGCTGCCTATGTGTA	NM_001396039.1
PFKL rev	TCAGAGGAGTAGAGGTTGT	
PFKM fwd	TCACCAACCTCTGCGTCATC	NM_204223.2
PFKM rev	TGTTCAGGTGGCTCGACTTC	
PKM2 fwd	CAGACCTGTGGCTATTGC	NM_205469.2
PKM2 rev	CATTGTCCAGCGTCACTT	
LDHA fwd	CGTCAGCAAGAAGGAGAA	NM_205284.2
LDHA rev	AGCCACTACCGATAACAC	
MYC fwd	GAAGCGAACGAGTCTGAA	NM_001030952.2
MYC rev	AGTTGTGTTGGTGGATGTT	
ALV-J *env* fwd	TGCGTGCGTGGTATTATTTC	GU982308.1
ALV-J *env* rev	AATGGTGAGGTCGCTGACTGT	
GAPDH fwd	GAGAAACCAGCCAAGTATGA	NM_204305.2
GAPDH rev	CTGGTCCTCTGTGTATCCTA	
CCL4 siRNA 1 S CCL4 siRNA 1 AS CCL4 siRNA 2 S CCL4 siRNA 2 AS CCL4 siRNA 3 S CCL4 siRNA 3 AS	CUGCUGCACCACUUACAUA UAUGUAAGUGGUGCAGCAG CAAAGCCUGCCAUCAUCUU AAGAUGAUGGCAGGCUUUG CAGCACAUAUAGCUCGACA UGUCGAGCUAUAUGUGCUG	NM_204720.3

### Protein extraction and immunoblotting

Whole-cell lysates were prepared with Cell Lysis Buffer (Cell Signalling Technologies, USA), separated by 10% SDS–PAGE at 120 V for 90 min, and transferred to polyvinylidene difluoride membranes at 50 V for 150 min. The membranes were blocked in TBS-T containing 5% nonfat dry milk (Bio-Rad, California, United States). Primary antibodies were incubated overnight at 4°C with agitation. The following antibodies were used to determine protein expression: rabbit polyclonal antibody against HK1 (A1054, Abclonal), rabbit polyclonal antibody against HK2 (A0994, Abclonal), rabbit polyclonal antibody against PKM2 (K001645P, Solarbio), rabbit monoclonal antibody against actin (ab179467, Abcam), mouse monoclonal antibody against c-Myc (ab56, Abcam), rabbit polyclonal antibody against LDHA (K002251P, Solarbio) and mouse monoclonal antibody against JE9, which is specific to the envelope protein of ALV-J. After washing extensively with TBST, the membranes were incubated with secondary antibodies (anti-rabbit or anti-mouse horseradish peroxidase conjugate) for 1 h at room temperature. After extensive washing with TBST, the blots were developed using enhanced chemiluminescent detection reagents on a FluorChem Q imaging system (Protein Simple, United States).

### Statistical analyses

The statistical analysis was performed with the Statistical Package for the Social Sciences (version 16.0) software. Statistical significance was assessed using a two-tailed unpaired Student’s *t*-test with a *p* value threshold of <0.05. * represents fold change ≥2 and *p* < 0.05; ** represents fold change ≥2 and *p* < 0.01.

## Results

### CCL4 is negatively regulated by glucose metabolism in chicken macrophages

**Figure 1:**
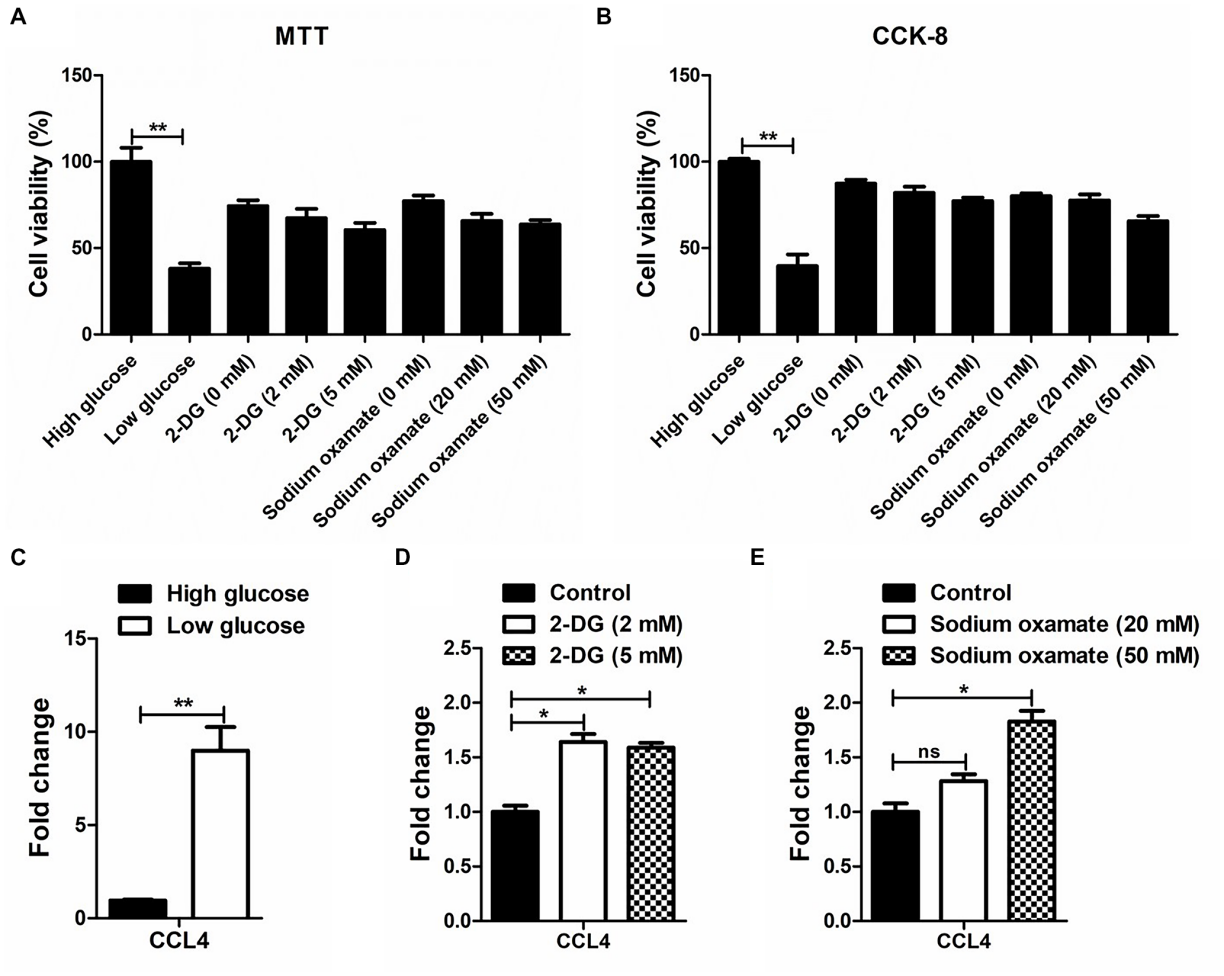
The response of CCL4 on glucose metabolism in chicken HD11 cells. MTT **(A)** and CCK-8 **(B)** assay were performed to evaluate the cytotoxicity on HD11 cells of treatment with high or low glucose, the glycolytic inhibitor 2-DG treatment and the LDHA inhibitor sodium oxamate for 48 h. **(C)** Relative expression analysis of chicken CCL4 in HD11 cells treated with high or low glucose for 48 h. **(D)** Relative expression analysis of chicken CCL4 in HD11 cells treated with the glycolytic inhibitor 2-deoxy-D-glucose (2-DG) for 48 h. **(E)** Relative expression analysis of chicken CCL4 in HD11 cells treated with the specific LDHA inhibitor sodium oxamate for 48 h. Error bars represent the s.d., *n* = 3. *p < 0.05 and **p < 0.01 (two-tailed Student’s *t*-test).

MTT and CCK-8 assays were performed to analyze the influence of glucose and the glycolytic inhibitor on the proliferation of the chicken macrophage cell line HD11. As shown in Figures 1A,B, low glucose-treated HD11 cells showed a lower cell viability rate than the high glucose-treated HD11 cells at 48 h. Treatment with low concentrations of glycolytic inhibitors (2-deoxy-D-glucose and sodium oxamate) did not significantly alter the viability of HD11 cells. We further found that in contrast to high glucose treatment, low glucose treatment significantly (*p* < 0.01) upregulated CCL4 expression in HD11 cells (Figure 1C). Consistent with this finding, the glycolytic inhibitor 2-deoxy-D-glucose, which targets hexokinase, the entry-point enzyme for glycolysis, also induced CCL4 expression (Figure 1D). We next blocked LDHA activity with the specific LDHA inhibitor sodium oxamate, and found that treatment with sodium oxamate also enhanced CCL4 expression in HD11 cells (Figure 1E). These results indicate that CCL4 is negatively regulated by glucose metabolism.

### ALV-J infection inhibited CCL4 expression in chicken macrophages

**Figure 2:**
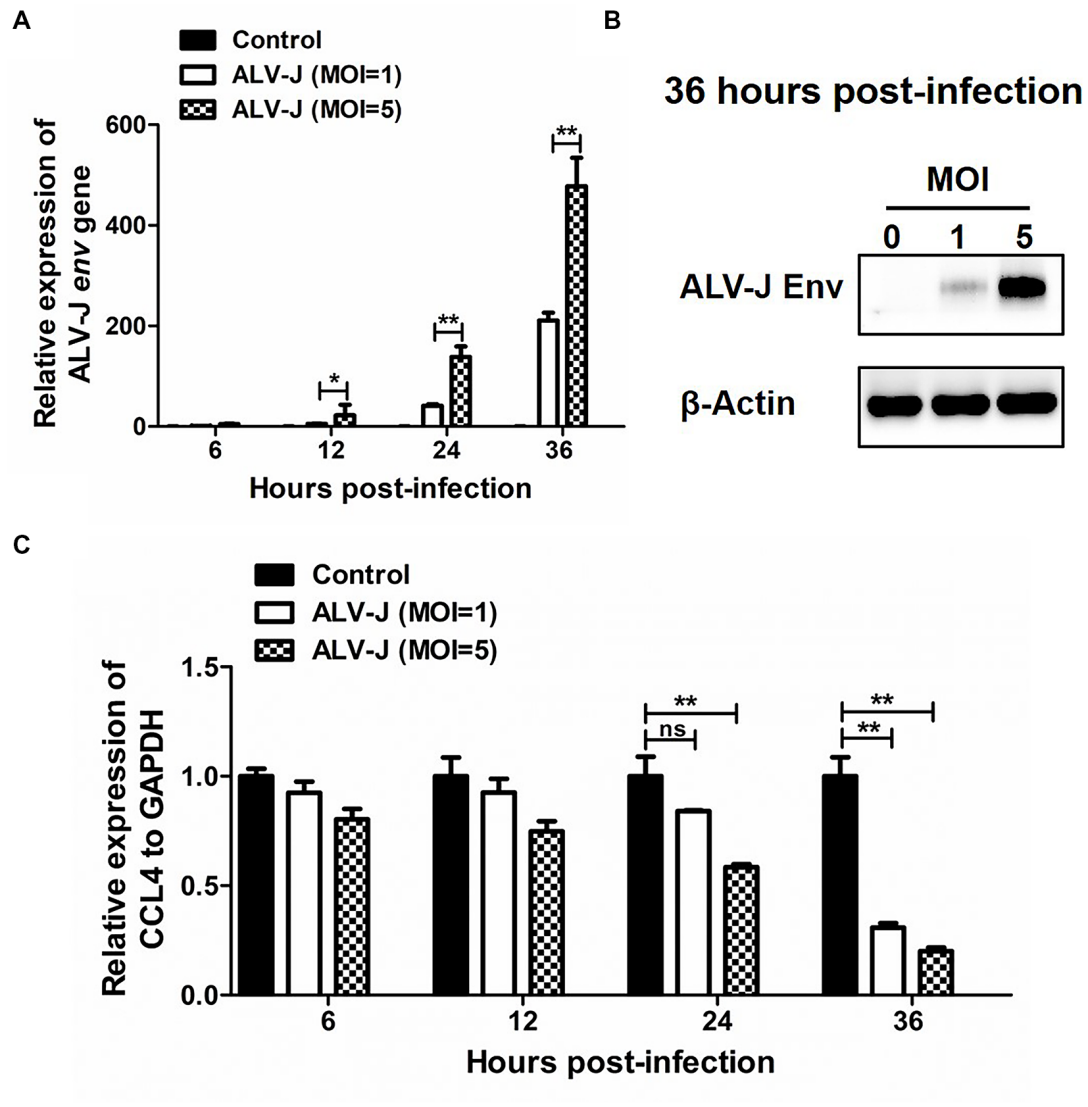
The expression of CCL4 in response to ALV-J infection in chicken HD11 cells. RT–qPCR **(A)** and Western blotting **(B)** analysis of ALV-J *env* gene expression in HD11 cells at different time points of ALV-J infection. **(C)** RT–qPCR analysis of the chicken CCL4 gene in HD11 cells at 6, 12, 24, and 36 hpi. Error bars represent the s.d., *n* = 3. *p < 0.05 and **p < 0.01 (two-tailed Student’s *t*-test).

We next investigated the dynamic response pattern of the chemokine CCL4 in chicken macrophages infected with the ALV-J strain JS09GY3. We first observed that the mRNA expression level of the ALV-J *env* gene gradually increased from 6 to 36 hpi in the chicken macrophage cell line HD11 infected with ALV-J at MOIs of 1 and 5 (Figure 2A). The protein expression level of the ALV-J *env* gene was increased in HD11 cells infected with ALV-J for 36 h at an MOI of 5, which further confirmed that ALV-J successfully infected HD11 cells (Figure 2B). The results showed that ALV-J infection persistently reduced the expression of the CCL4 gene in HD11 cells from 6 to 36 hpi. In particular, the expression levels of the CCL4 gene were significantly (*p* < 0.01) downregulated in ALV-J-infected HD11 cells at 24 and 36 hpi (Figure 2C). Compared with a low MOI of ALV-J, CCL4 expression was more significantly reduced at a high MOI of 5. These results showed that ALV-J infection inhibited chemokine CCL4 expression in chicken macrophages.

### ALV-J envelope protein inhibit CCL4 expression in chicken macrophages

**Figure 3:**
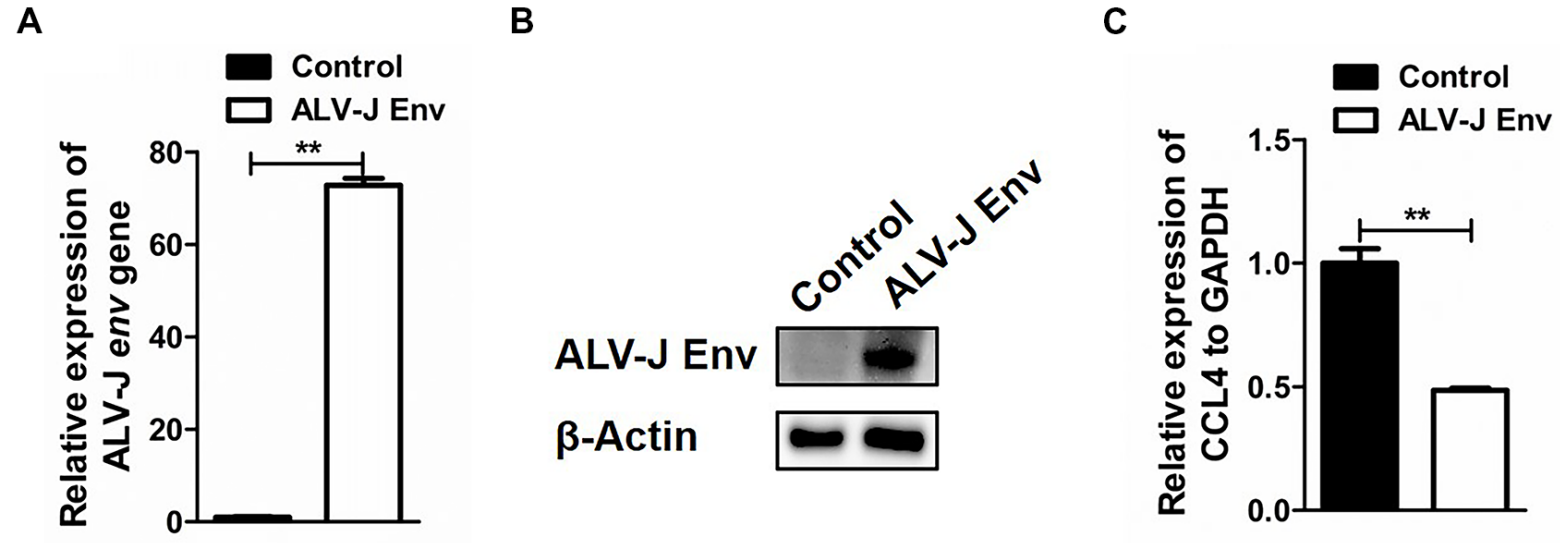
Overexpression of the ALV-J *env* gene inhibited the expression of the CCL4 gene in chicken cells. RT–qPCR **(A)** and Western blotting **(B)** analysis of ALV-J *env* gene in HD11 cells transfected with pcDNA3.1-ALV-J Env or the control plasmid for 48 h. **(C)** RT–qPCR analysis of the CCL4 gene in HD11 cells transfected with pcDNA3.1-ALV-J Env or the control plasmid for 48 h. Error bars represent the s.d., *n* = 3. **p < 0.01 (two-tailed Student’s *t*-test).

To investigate the specific mechanism by which ALV-J infection inhibits CCL4 expression, HD11 cells were transfected with the plasmid pcDNA3.1-ALV-J Env for 48 h, and then the expression of CCL4 was assessed by RT–qPCR. As shown in Figures 3A,B, ALV-J Env mRNA and protein were abnormally expressed in HD11 cells transfected with the plasmid pcDNA3.1-ALV-J Env compared to the control. Consistent with ALV-J infection, overexpression of ALV-J Env also significantly (*p* < 0.01) inhibited the expression of CCL4 expression in HD11 cells (Figure 3C). These results suggested that the ALV-J-encoded envelope protein is responsible for CCL4 inhibition.

### CCL4 Regulates glucose metabolism in chicken macrophages

**Figure 4:**
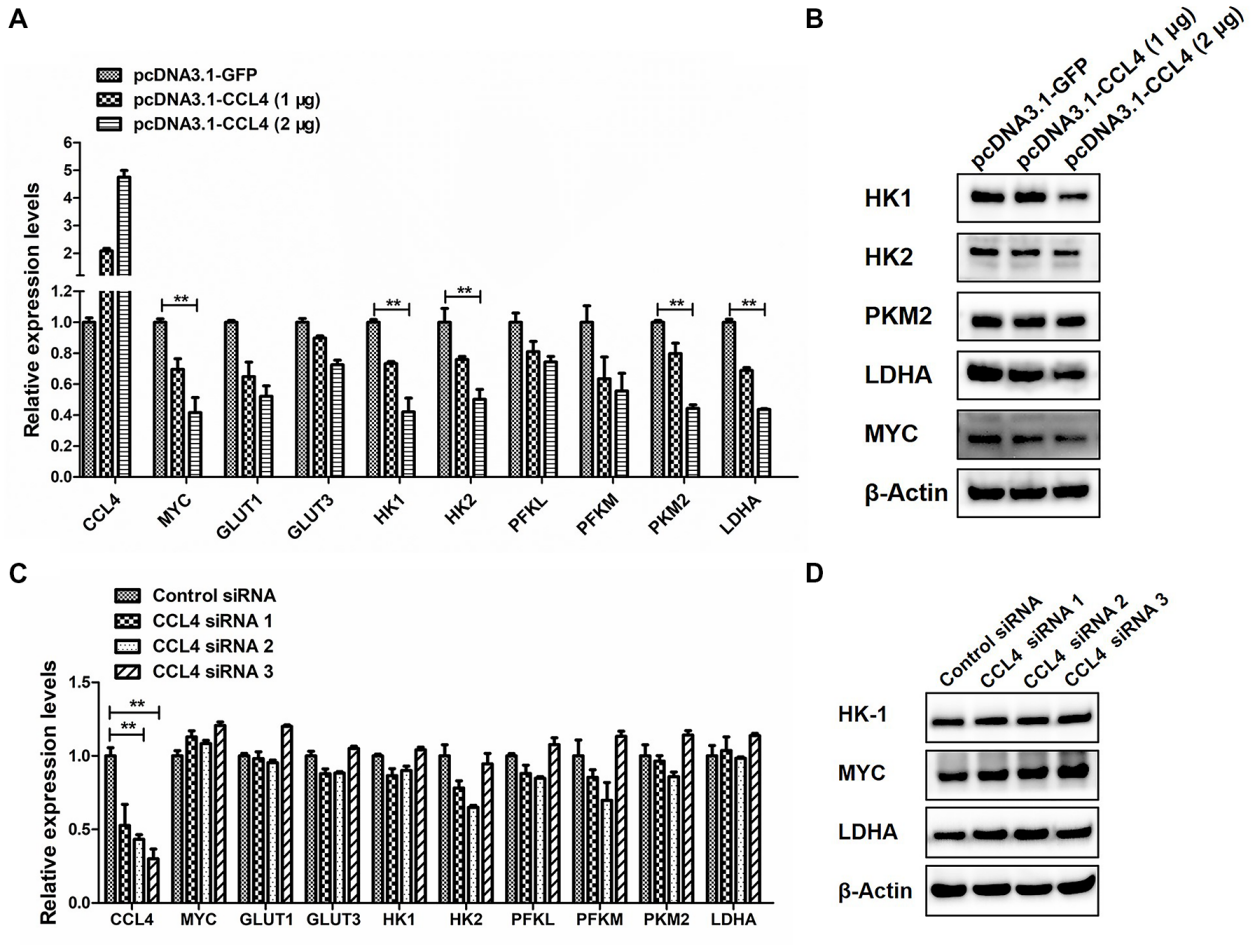
CCL4 regulates the expression of glucose metabolism genes. **(A)** Relative expression analysis of the chicken CCL4, GLUT1, GLUT3, HK1, HK2, PFKM, PFKL, PKM2, LDHA, and c-myc genes in HD11 cells transfected with pcDNA3.1-CCL4 or the control plasmid for 48 h. **(B)** Western blotting analysis of HK1, HK2, PKM2, MYC, and LDHA gene expression in HD11 cells transfected with pcDNA3.1-CCL4 or the control plasmid for 48 h. **(C)** Relative expression analysis of the chicken CCL4, GLUT1, GLUT3, HK1, HK2, PFKM, PFKL, PKM2, LDHA, and c-myc genes in HD11 cells transfected with the control or chicken CCL4 siRNA for 48 h. **(D)** Western blotting analysis of HK1, MYC, and LDHA gene expression in HD11 cells transfected with the control or chicken CCL4 siRNA for 48 h. Error bars represent the s.d., *n* = 3. **p < 0.01 (two-tailed Student’s *t*-test).

We then assessed the influence of CCL4 on glucose metabolism. As shown in Figure 4, overexpression of CCL4 inhibited the expression of glucose metabolism-related genes in HD11 cells in a dose-dependent way. Transfection of HD11 cells with the vector containing CCL4 for 48 h led to an obvious reduction in the mRNA levels of glucose metabolism-related genes, including MYC; glucose transporter 1 (GLUT1); GLUT3; hexokinase 1 (HK1); HK2; phosphofructokinase, liver type (PFKL); phosphofructokinase, muscle type (PFKM); pyruvate kinase M2 (PKM2) and LDHA (Figure 4A). In particular, CCL4 significantly (*p* < 0.01) downregulated the expression of the key genes HK1, HK2, PKM2, MYC, and LDHA, which regulate glycolysis at the protein level in HD11 cells (Figure 4B). Knockdown of chicken CCL4 gene by RNAi did not change the mRNA levels of glucose metabolism-related genes, including MYC, GLUT1, GLUT3, HK1, HK2, PFKL, PFKM, PKM2, and LDHA in HD11 cells (Figure 4C). However, CCL4 knockdown significantly raised the protein expression levels of HK1, MYC, and LDHA in HD11 cells (Figure 4D). These results suggested that CCL4 inhibits glucose metabolism in chicken macrophages.

### CCL4 Mediates ALV-J replication by regulating the expression of glucose metabolism genes in chicken macrophages

**Figure 5:**
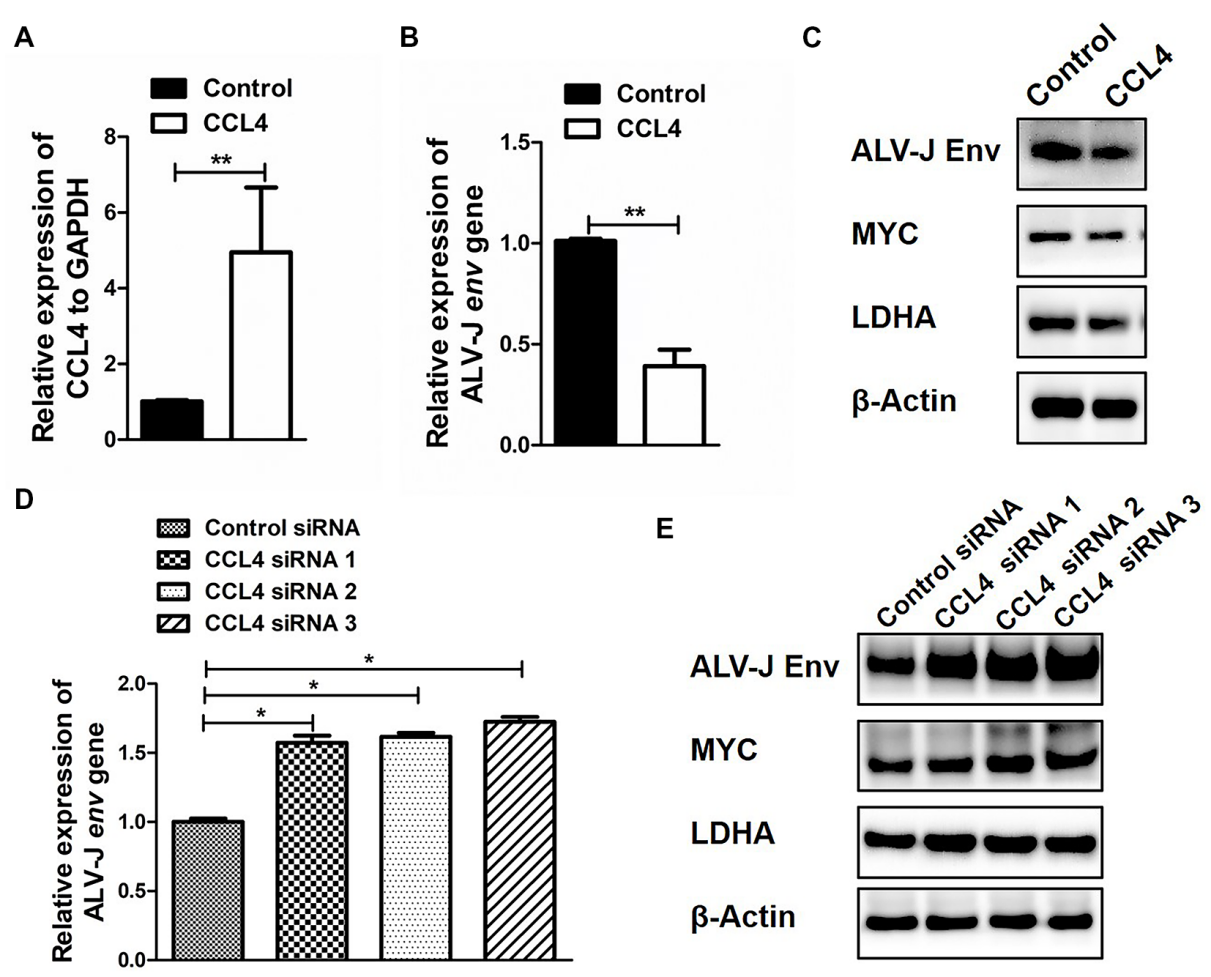
CCL4 regulates the replication of ALV-J and the expression of glucose metabolism genes. RT–qPCR analysis of CCL4 **(A)** and ALV-J *env* **(B)** gene expression in HD11 cells, which were first infected with the ALV-J virus at an MOI of 5 and then transfected with pcDNA3.1-CCL4 or the control plasmid for 48 h. **(C)** Western blotting analysis of ALV-J *env*, MYC, and LDHA gene expression in HD11 cells were first infected with the ALV-J virus at an MOI of 5 and then transfected with pcDNA3.1-CCL4 or the control plasmid for 48 h. **(D)** RT–qPCR analysis of ALV-J *env* gene expression in HD11 cells was first infected with the ALV-J virus at an MOI of 5 and then transfected with the control or chicken CCL4 siRNA for 48 h. **(E)** Western blotting analysis of ALV-J *env*, MYC, and LDHA gene expression in HD11 cells, which were first infected with the ALV-J virus at an MOI of 5 and then transfected with the control or chicken CCL4 siRNA for 48 h. Error bars represent the s.d., *n* = 3. **p < 0.01 (two-tailed Student’s *t*-test).

Next, we further investigated the influence of CCL4 on the reprogramming of glucose metabolism after ALV-J infection in chicken macrophages. HD11 cells were transfected with CCL4 after ALV-J infection, and the impact of CCL4 on ALV-J replication proliferation was assessed. Compared with the control group, transfection of CCL4 significantly (*p* < 0.01) inhibited the expression of the ALV-J *env* gene at both the mRNA and protein levels in HD11 cells (Figures 5A–C). We found that CCL4 substantially repressed the protein expression of two key genes, c-myc and LDHA, that regulate glycolysis in ALV-J-infected HD11 cells. Knockdown of the chicken CCL4 gene by RNAi in HD11 cells was performed further to confirm the role of chicken CCL4 in antiviral defense. Conversely, CCL4 knockdown significantly (*p* < 0.05) increased the expression of the ALV-J *env* gene at both the mRNA and protein levels in HD11 cells (Figures 5D,E). Moreover, CCL4 knockdown notably increased the protein expression of two key genes, c-myc and LDHA, that regulate glycolysis in ALV-J-infected HD11 cells (Figure 5E). These findings indicate that the CCL4 gene possesses an antiviral function in ALV-J infection by interfering with the reprogramming of glucose metabolism after ALV-J infection.

## Discussion

Recently, the field of immunometabolism has made great strides to unveil the crucial role of intracellular metabolism in regulating immune cell function (Lercher et al., 2020). Activation of immune cells engages specific metabolic pathways and rearranges their oxidation–reduction (redox) system, which supports metabolic reprogramming (Muri and Kopf, 2021). In the present study, we described a role for the chemokine CCL4 as a regulator of macrophage glucose metabolism and identified this as an antiviral mechanism modulating metabolism during ALV-J infection. CCL4 is negatively regulated by ALV-J infection and glucose metabolism. Therefore, the interaction between the innate immune system and glucose metabolism may be involved in ALV-J infection. Furthermore, regulation of glucose metabolism may be an effective strategy to limit ALV-J infection.

As an important player in the antiviral response, interferons have been demonstrated to modulate macrophage metabolism. For example, IFN-γ-mediated metabolic reprogramming is a key component of classical inflammatory macrophage activation (Su et al., 2015). IFN-γ treatment restrains host cell glycolysis, which is accompanied by a reduction of glucose transporter-1 (GLUT1) and hypoxia-inducible factor-1α (HIF-1α) expression (Shima et al., 2018). One recent study has also shown that type I interferons restrain macrophage metabolism, and IFN-β prevents the shift to aerobic glycolysis in inflammatory macrophages (Olson et al., 2021).

Our results show that CCL4 is a key factor limiting the replication of ALV-J in chicken macrophages. In response to ALV-J infection, the expression level of the CCL4 gene was significantly increased in the bursa of Fabricius of 10-day-old chickens. In contrast, this increased expression of CCL4 was subsequently reduced with increasing levels of viral infection in 30-day-old chickens (Hang et al., 2014). This study found that ALV-J infection *in vitro* significantly reduced CCL4 gene expression in chicken macrophages, especially at high MOI. We further confirmed that the ALV-J envelope protein is responsible for CCL4 inhibition. Furthermore, CCL4 significantly inhibited the expression of the ALV-J envelope (*env*) gene at both the mRNA and protein levels in chicken macrophages. A recent study showed that the antiviral actions of type I interferons are mediated through the CCL4-dependent recruitment of inflammatory monocytes rather than through the direct inhibition of virus replication *via* the action of intracellular interferon-stimulated genes (Parekh et al., 2019). Thus, these results indicated that ALV-J infection might resist the chemokine-mediated antiviral response by inhibiting the expression of CCL4. Inhibition of glucose metabolism may be an essential mechanism by which CCL4 inhibits the replication of ALV-J in chicken macrophages.

The chemokine CCL4 is necessary for the control of viral infection. However, this chemokine’s effect on glucose metabolism has not been thoroughly investigated. The present study has uncovered a missing link between the chemokine CCL4 and glucose metabolism in the context of ALV-J infection and glucose treatment. Using two models (glucose treatment and virus infection), we revealed that the chemokine CCL4 was affected by glucose metabolism and ALV-J infection. We then confirmed that CCL4 is a negative regulator of glucose metabolism by overexpression of CCL4. Significantly, CCL4 inhibits ALV-J-mediated reprogramming of glucose metabolism in chicken macrophages. One recent study also showed that CCL4 plays a significant role in glucose metabolism and is upregulated in diabetes mellitus (Chang and Chen, 2021). CCL4 may be involved in glucose metabolism through its C-C chemokine receptor 5 (CCR5) receptor. It has been reported that the chemokine receptor CCR5 specifically inhibits aerobic glycolysis in memory-like CD4+ T cells (Blanco et al., 2021). CCR5 also regulates insulin signaling in the hypothalamus and contributes to systemic insulin sensitivity and glucose metabolism (Chou et al., 2016). Studies have also demonstrated that another ligand of CCR5, CCL5, is critical for glucose metabolism (Chou et al., 2016; Gao et al., 2016; Ajoy et al., 2021). Therefore, regulating glucose metabolism by CCL4 and its receptor CCR5 may be an effective strategy to limit ALV-J infection.

Vaccines, chemicals, and antibiotics generally mediate poultry disease prevention and control. However, there is currently no effective commercial vaccine for ALV-J. It is relatively challenging to develop vaccines owing to several reasons: ALV-J displays a high level of genetic variation and recombination, posing the potential for generating novel strains (Xu et al., 2022). The second is that ALV-J has evolved varied strategies to evade or suppress host immune response (Mo et al., 2021; Wang et al., 2022; Xu et al., 2022). In addition, it is difficult to induce the production of ALV-J-specific antibodies for uncertain reasons and thus leads to great difficulties in ALV-J vaccine research. Moreover, the extensive use of antibiotics and chemicals in livestock has resulted in environmental and human health concerns, particularly concerning the emergence of drug-resistant bacteria in the food chain (Lowenthal et al., 1999). Cytokines are crucial immune system regulators, and cytokine therapy is considered a natural alternative for disease control (Lowenthal et al., 1999, 2000). Cytokines contribute a central role in disease control in a variety of diseases (Stokkers and Hommes, 2004), including avian diseases (Muri and Kopf, 2021). CCL4 is an excellent candidate as a therapeutic agent and adjuvant due to its immunotherapeutic properties against ALV-J infection. Therefore, applying and integrating CCL4 may provide and open new horizons to control and prevent ALV-J.

Chicken macrophage-like HD11 cells are an established chicken myelomonocytic cell line. This cell line is derived from chicken bone marrow and transformed by the v-myc encoding retrovirus MC29 (Beug et al., 1979). HD11 cells are normally loosely adherent to plastic and have an ovoid shape (Wisner et al., 2011), and they are used as an important immune cellular model in poultry to study the immune response and antiviral immunity of ALV-J. In this study, we confirmed the antiviral effect of the chemokine CCL4 on ALV-J infection in HD11 cells. The previous study showed that increased expression of CCL4 was associated with ALV-J infection in the bursa of Fabricius of chickens (Hang et al., 2014). Other studies also showed that CCL4 involved in ALV-J infection (Chen et al., 2018; Ruan et al., 2021). These findings may be indicated that CCL4 may have clinical significance for promotion of ALV-J infection. As a small signaling molecule, CCL4 might be become a potential drug target for developing antiviral agents for ALV-J infection. For example, CCL4 agonists could be the most promising as anti-ALV-J drugs. In addition, we also need to consider that CCL4 agonists may cause tissue damage caused by a storm of inflammatory factors.

In conclusion, the present study shows that the chemokine CCL4 regulates macrophage glucose metabolism and exerts antiviral protective effects by inhibiting glucose metabolism. These findings might provide an important mechanistic insight into the metabolic response of macrophages to avian tumor virus infection. Further investigating the metabolic effects of chemokine metabolic effects on macrophages will help clarify chemokines’ complex beneficial and detrimental roles in antiviral innate immunity and incorporate molecular docking and interdisciplinary networking approaches to develop effective vaccines to combat ALV-J infection in chickens.

## Data availability statement

The raw data supporting the conclusions of this article will be made available by the authors, without undue reservation.

## Ethics statement

The animal study was reviewed and approved by the Committee approved the protocol on the Ethics of Animal Experiments of Yangzhou University (license number: 06R015).

## Author contributions

HC, QX, and XH: conceptualization. GZ, HW, HL, TW, WG, SZ, CW, and WC: methodology. HL, GZ, and HW: software, writing—original draft preparation, and visualization. XH, HC, CW, and QX: validation. XH and HC: resources, and supervision and project administration. HC and QX: funding acquisition. All authors contributed to the article and approved the final manuscript.

## Funding

This study was supported by the National Natural Science Foundation of China (31602032, 81773013, and 91540117), the Key Program of Changzhou Science and Technology Bureau (CE20202033), and the Priority Academic Program Development of Jiangsu Higher Education Institutions (Animal Science and Veterinary Medicine).

## Conflict of interest

The authors declare that the research was conducted in the absence of any commercial or financial relationships that could be construed as a potential conflict of interest.

## Publisher’s note

All claims expressed in this article are solely those of the authors and do not necessarily represent those of their affiliated organizations, or those of the publisher, the editors and the reviewers. Any product that may be evaluated in this article, or claim that may be made by its manufacturer, is not guaranteed or endorsed by the publisher.
